# A qualitative exploration of STI partner notification services delivery models among key stakeholders in rural southwestern Uganda

**DOI:** 10.3389/frph.2025.1564836

**Published:** 2025-07-07

**Authors:** Pooja Chitneni, Moran Owembabazi, Eunice Kanini, Rosemary Kansiime, Winnie R. Muyindike, Christina Psaros, Jessica E. Haberer, Lynn T. Matthews

**Affiliations:** ^1^ Division of Global Health Equity and General Internal Medicine, Brigham and Women’s Hospital, Boston, MA, United States; ^2^ Harvard Medical School, Boston, MA, United States; ^3^ Mbarara Regional Referral Hospital and Mbarara University of Science and Technology, Mbarara, Uganda; ^4^ Department of Psychiatry, Behavioral Medicine Program, Massachusetts General Hospital, Boston, MA, United States; ^5^ Center for Global Health, Massachusetts General Hospital, Boston, MA, United States; ^6^ Division of Infectious Diseases, Heersink School of Medicine, University of Alabama at Birmingham, Birmingham, AL, United States

**Keywords:** sexually tranmistted infections, sub-Sahara Africa (SSA), Uganda, partner notification services, task shifting

## Abstract

**Background:**

Comprehensive STI care requires not only patient treatment but STI partner notification (PN) and evaluation to prevent ongoing STI transmission and reinfection. In rural, southwestern Uganda, we explored healthcare practitioners’ views on three STI PN models that focused on task-shifting to non-physician practitioners to inform the development of a novel STI PN services delivery system.

**Methods:**

From September to November 2023, we conducted individual in-depth interviews with 32 participants from 4 categories (8 participants from each category): patients with a self-reported history of having an STI in Uganda, healthcare workers (physicians, nurses, and community health workers), pharmacists, and healthcare administrators (Ministry of Health officials and regional referral hospital administrators). Interviews explored participants’ views on a nursing-based, pharmacy-based, and community-based STI PN models as well as healthcare system tools and needs to facilitate PN. We used inductive and deductive approaches to generate a codebook guided by the Consolidated Framework for Implementation Research in a thematic analysis.

**Results:**

Ten female and twenty-two male participants participated in individual in-depth interviews. The median age of the patient and healthcare practitioner participants was 32 and 34 years, respectively. We found that (1) the nursing model was overall supported as nurses (though with one participant noting dissatisfaction with nurses), (2) pharmacies are well-positioned to engage in STI PN as they are early points of contact for patients, incentivized monetarily by PN and patient-delivered partner medication, and have the potential to physically restructure to create private spaces and increase counseling training, (3) the community-based model should center on village health teams and focus on advocacy and education.

**Conclusion:**

Given the high STI incidence globally and in sub-Saharan Africa, exploring innovative STI care models that resonate with patients and healthcare practitioners will be important. Future work includes a Delphi method to refine these models for testing.

## Introduction

The global sexually transmitted infection (STI) burden is high. In 2020, the global incidence of four common curable STIs including chlamydia, gonorrhea, trichomoniasis, and syphilis was estimated to be one million each day (1). Per the World Health Organization (WHO), the African Region has the highest STI burden, with an estimated 96 million STIs diagnosed in 2020 (1).

Reasons for increasing STI trends are likely multifactorial. In most resource-limited settings, STI diagnostics are unavailable. Instead, providers care for STIs through syndromic management, or the diagnosis of STI syndromes based on clinical signs and symptoms resulting in both over and under-diagnosis of STIs (2). Additionally, comprehensive STI care requires not only patient treatment but also STI partner notification (PN) and evaluation to prevent ongoing STI transmission and post-treatment reinfection. STI PN is recommended by the World Health Organization and the Ugandan ministry of health (3, 4). However, many settings lack the ability to support PN and evaluation, which could contribute to high STI rates.

Despite the importance of STI PN, few methods and tools exist to support this complex process. There are three main methods for PN: provider referral, where a healthcare provider informs a partner of a potential STI exposure; patient referral, where the patient notifies their partner; and contract-referral, where the provider and patient agree on a timeframe for the partner to attend the clinic for evaluation, after which the provider may contact the partner directly if they have not sought treatment (5). Other tools to support STI PN include STI PN notes, in which a clinic writes a note to partners outlining a potential STI exposure and the need for clinic evaluation and/or medication, and patient-delivered partner medication (PDPM), which allows clinicians to give medications and/or prescriptions to patients to deliver to their partners for presumptive STI treatment.

While the above-mentioned STI PN methods and tools are utilized to varying degrees in resource-rich settings, clinicians often default to patient-based STI PN in resource-limited settings (RLS) due to a lack of healthcare resources. Studies suggest that provider referral results in increased STI PN with a systematic review of STI PN in sub-Saharan Africa demonstrating 25% of partners sought evaluation after patient referral compared to 69% of partners after provider referral (6). Additionally, prior qualitative work in our study setting in rural Uganda demonstrates that patients managing STIs prefer provider-assistance in STI PN (7). However, we know little about how to design or implement STI PN methods and tools in RLS.

**Figure 1:**
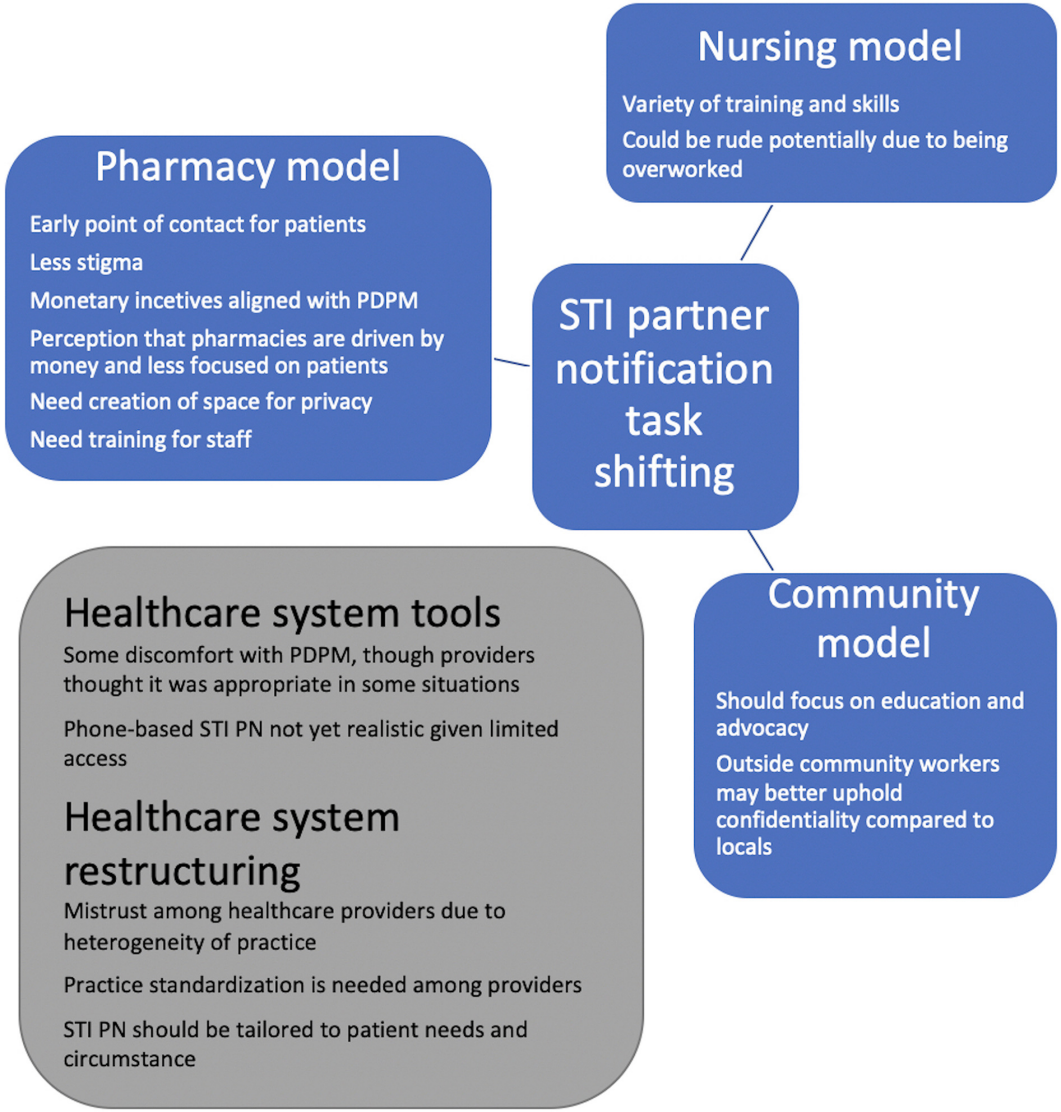
Graphic of candidate STI partner notification services delivery model themes.

Inspired by the concept of task-shifting from the HIV literature in RLS, we developed three STI PN models that focused on non-physician healthcare providers with the idea of developing a novel STI PN services delivery system. We conducted in-depth qualitative interviews with relevant stakeholders including healthcare workers with experience working in the field of STIs (physicians, nurses, lab technicians), pharmacists, healthcare administrators, and people who have had STIs in rural, southwestern Uganda. We explored integrating STI PN into existing healthcare structures and systems guided by the Consolidated Framework for Implementation Research (CFIR) (8). We focused on a nursing-based STI PN model, a pharmacy-based STI PN model, and a community-based STI PN model (Figure 1). These three settings have providers who routinely counsel patients (with the appropriate resources) and who are already heavily involved in HIV care in many settings.

## Methods

### Study setting

Study methods are reported following the COREQ guidelines (9). The study took place within Mbarara Regional Referral Hospital (MRRH), one of 14 academic regional referral hospitals in the country. Mbarara is a small city located in southwestern Uganda, approximately 290 km southwest of Uganda's capital, Kampala. This hospital cares for both inpatient and outpatient, with several specialties providing outpatient care, including primary care, HIV, and OB-Gyn clinics.

### Study participants

We used purposive sampling to recruit 32 participants from 4 categories (8 participants from each category): patients with a self-reported history of having an STI in Uganda, healthcare workers (including physicians, nurses, and community health workers), pharmacists, and healthcare administrators (including Ministry of Health officials and regional referral hospital administrators). Individual patient participants were recruited from academic medical center clinics through referrals from healthcare providers. Healthcare provider participants were recruited with the goal of having representation among an array of fields, and among those working in the public and private healthcare sectors. Participants were interviewed from September 2023 to November 2023. For the purposes of maintaining anonymity, participants were referred to their overall category (i.e., healthcare worker) as opposed to their specific job (i.e., nurse).

Healthcare personnel inclusion criteria encompassed currently working in the outlined field, having relevant expertise related to STIs, and being age 18 years or greater. Patient inclusion criteria encompassed having a history of STI, being a patient at MRRH, and being age 18 years or greater. Exclusion criteria for all participants encompassed an inability to speak the local language (Runyankole) or English or provide informed consent (e.g., intoxication).

### Data collection

We developed semi-structured individual, in-depth interview guides to exploring participants' thoughts on STI PN methods. Separate interview guides were created for patient participants and provider participants. Questions explored participant thoughts on potential models and methods related to STI PN. We explored three STI PN models (nursing-based, pharmacy-based, and community-based) that focused on task-shifting, or redistributing healthcare tasks, to non-physicians with the goal of increasing efficiency while maintaining quality care. These models borrowed from literature related to HIV care task-shifting from clinicians to additional healthcare workers (10). In addition, questions focused on specific STI PN methods, such as PN notes, text messaging apps, and PDPM. The CFIR guided question development (Table 1) (8). This framework outlines five major domains to organize implementation research across various contexts, including intervention characteristics, the outer setting, the inner setting, characteristics of the individuals, and the implementation process. Additionally, a brief interviewer-administered questionnaire captured socio-demographic information.

**Table 1:** Consolidated framework for implementation research (CFIR) domains correlated with sample in-depth interview questions.

CFIR domains	Example in-depth interview questions
Characteristics of new technologies	What are your thoughts on patient delivered partner medications, or the idea of a patient delivering STI medications that they receive from clinic to take to their partner without the partner being evaluated.
Individual characteristics	What would help patients participate in this STI partner notification system?
Inner setting	How do different clinics, pharmacies, and community settings currently interact in regards to STI care overall and specifically STI partner notification?
Outer setting	Is there a role for STI partner notification efforts outside of the traditional healthcare system?
Implementation process	How could community healthcare workers support STI partner notification?

Two female Ugandan research assistants, trained in qualitative research methods and fluent in Runyankole and English, interviewed and digitally recorded participant interviews in Runyankole. These same interviewers then transcribed and translated the audio recordings to English. Interviews lasted between approximately 30–60 min and were conducted in a private research room on the Mbarara University of Science and Technology campus.

### Analysis

The analytic team consisted of a clinician-scientist (PC) with a medical doctoral degree from the United States, a public health researcher (MO) with a master's in public health from Uganda, a nurse-researcher (RK) with a nursing degree from Uganda, and a researcher (EK) with an procurement degree from Uganda. All team members had qualitative methods training and had worked together on the topic of HIV and/or STI disclosure and PN.

We used deductive and inductive approaches in a thematic analysis of the interviews (11). One team member created initial codes in a deductive manner based on the interview guide, the CFIR, and the STI PN literature (8, 12). Four team members reviewed the transcripts to iteratively revise the codebook in an inductive manner based on open-coding to identify emergent codes. Three team members double-coded several transcripts meeting with the entire team to discuss each of these transcripts in detail and resolve disagreements. Two team members then coded the remaining transcripts independently with continued team meetings to further refine codes and develop themes. The team generated themes through iterative discussions on emerging patterns. The preliminary themes were shared during a group meeting with 25 members from the representative groups (i.e., peer navigators, healthcare workers, pharmacists, healthcare administrators, and community health workers to confirm that our team's interpretations were appropriate and harmonized with their thoughts and experiences. No additional changes were made to the themes after this meeting.

Qualitative data were managed and analyzed with the aid of NVivo 1.6.2 software. Quantitative data were analyzed descriptively using STATA 15.1 software.

### Ethics

Study procedures were approved by the Mbarara University of Science and Technology Research Ethics Committee (Protocol MUST-2022-580), the Uganda National Council of Science and Technology, and the Mass General Brigham in Boston, MA, USA (Massachusetts General Hospital and Brigham and Women's Hospital) Institutional Review Board (Protocol 2022P003019).

## Results

### Participant characteristics

We conducted 32 individual in-depth semi-structured qualitative interviews with 10 female participants and 22 male participants (Tables 2, 3). The median age of the patient and healthcare provider participants was 32 and 34 years, respectively. Three-quarters of the patient participants were living with HIV, most 5/8 (63%) reported ever notifying a partner of an STI, and 1/8 (13%) reported partner treatment. Among healthcare provider participants, 8/24 (33%) were pharmacists, 3/24 (13%) each were community health workers, nurses, hospital administrators, and district health administrators, and 2/24 (8%) each were doctors and ministry of health officials. The median amount of time participants worked in their current job was 5 years and in their current career field was 9 years.

**Table 2:** Patient participant demographics and STI characteristics.

Characteristic	Patient participants	
	*N*, Median	%, IQR
Age	32	31.5–36.5
Sex		
Men	4	50%
Women	4	50%
Participant HIV status		
Living with HIV	6	75%
Not living with HIV	1	13%
Unknown	1	13%
STI diagnosed		
Syphilis	6	75%
Gonorrhea	3	38%
Lifetime sex partners	5.5	2.5–13.5
Participant male condom use		
Always	1	13%
Sometimes	5	63%
Never	2	25%
Partner notification		
Yes	5	63%
No	3	38%
Partner treatment		
Yes	1	13%
No	6	75%
I don’t know	1	13%
History of transactional sex		
Yes	3	38%
No	5	63%
History of sexual assault		
Yes	2	25%
No	6	75%

**Table 3:** Healthcare provider participant demographics.

Characteristic	Healthcare provider participant	
	*N*, Median	%, IQR
Age	34	30–44
Sex		
Men	18	75%
Women	6	25%
Role		
Community health worker	3	13%
Nurse	3	13%
Doctor	2	8%
Pharmacist	8	33%
Hospital administrator	3	13%
District health administrator	3	13%
Ministry of health official	2	8%
Hospital setting		
Health center 4	2	8%
Regional referral hospital	8	33%
National referral hospital	1	4%
Not applicable	13	54%
Work sector		
Public	14	58%
Private, for-profit	8	33%
Private, non-profit	2	8%
Years working in current job	5	(2.75–13.5)
Years working in current field	9	(3.5–15)

#### Domaine 1: nursing-based STI PN model

*Theme 1: People generally trust and respect nurses to fulfill many roles.*

Participants found nurses to have a wide array of skills and were more familiar with nurses compared to other healthcare provider and/or models. Nurses were thought to be adept counselors who were well-suited for STI PN.

“Nurses are good and they have basic training in counseling, in medicine, and in general patient management. They study managing a patient right from reception up to follow-up so they have a very big stake…They interpret prescriptions, they dispense drugs, they do follow up and counsel.” Male, Healthcare administrator 1

Nurses were also felt to be professional and trustworthy with both patient and provider participants believing nurses would uphold the confidentiality of issues related to a potentially stigmatizing condition.

“They have the power to keep peoples’ secrets. When Nurses have confidentiality and treat patients well, the patients give them respect and they follow what they have been told.” Male, Healthcare worker 1

“Nurses are also advocates for patients. They have to advocate for better services and then they can also give health education to patients because they are also health educators.” Female, Healthcare worker 7

There was notably a dissenting voice who disliked some interactions with nurses and questioned their ability to keep things confidential.

“They [nurses] are rude, and they speak so badly. Most of the female nurses don’t keep the secrets you’re telling her. I really don’t support them.” Female, Patient 1

#### Domaine 2: pharmacy-based STI PN model

*Theme 1: Pharmacies are an early and appealing point of contact for patients seeking STI care.*

Participants outlined that patients often present to pharmacies as their first point of contact with the healthcare system. Even if not the first stop for participants, participants often seek medications directly from pharmacies for recurrent conditions. This approach allows patients to save time by avoiding long clinic queues, highlighting the role of pharmacies in providing convenient and accessible care.

“In a pharmacy setting, you have three sets of clients. The first is people who have gone to a clinic, they have a prescription of the medicine, and they are coming to buy their medicine. The next class is those who know that they have ever had such an infection and they know that they were given this or that, and they say ‘give me this medicine’ because the last time he went to hospital he had the same symptoms and signs. The other set is those that are first time health-care attention seekers. It’s their first time having an infection and they explain to you and they want the care but usually we want to first send them to a clinic or a lab for tests and then later get the medications.” Male, Pharmacist 1

Additionally, visiting certain clinics associated with curable STIs and/or HIV could be associated with perceived public stigma due to concerns of being observed by family, friends, or acquaintances. Because pharmacies treat many conditions of varying severity, and generally lack the stigma associated with certain clinics, they can be an appealing point of healthcare contact.

“Some people get symptoms…and he doesn’t care to know what disease he has. He will just run to the pharmacy because he or she doesn’t want to come here [the hospital] and get the real treatment which doesn’t cost anything. He or she rather looks at it as a shame for people to know that he is sick.” Female, Healthcare worker 2

*Theme 2: While pharmacies are not currently structured to support STI PN, with potential adaptations, they could take on this role.*

Most pharmacies lack the private spaces for patients to discuss sensitive topics preventing them from counseling on potentially stigmatizing conditions, like STIs. However, a few participants highlighted pharmacy models with private spaces and experience in counseling and mentioned the potential for expanding this design to more pharmacies.

“Because people who go to pharmacies are going to buy drugs the interface between them [pharmacy staff] and the patients is limited. Even the way they are being designed, it is usually one room, people get their drugs off the counter so the privacy to discuss private matters is compromised.” Female, Healthcare worker 3

Healthcare worker participants, were concerned that pharmacists didn't currently have the appropriate training or time to counsel patients on STI PN, though most participants stated that pharmacists would likely be interested in pursuing educational opportunities related to counseling. This interest was especially the case if trainings were financially supported. They also suggested that pharmacies could benefit from counselors sitting in the pharmacy to aid in STI PN.

“To me, I think we can train them [pharmacists] because knowledge is power so if it’s in the form of a bursary or studying a course to give them proper knowledge, then give them small pocket of a salary and take them for seminars too.” Male, Pharmacist 2

*Theme 3: Monetary incentives could align pharmacists with STI PN and PDPM.*

Healthcare worker and patient participants worried that pharmacists were motivated by financial interests alone, and that they were inappropriate to guide patients on treatment or partner notification/treatment options.

“Most of these pharmacists are business people, and people now go to them with a diagnosis they once had so they just go to buy it. The pharmacists can’t do much because they are after money, so I don’t think they can advise you or give you any counseling.” Male, Patient 2

Participants also realized that pharmacies are uniquely positioned to facilitate STI PN and potentially PDPM. Indeed, pharmacists could be incentivized to promote STI PN and encourage PDPM, as increased patient use of PDPM would result in increased sales of STI medications for pharmacies.

“Of course, making money is their primary objective. They need to make money, but partner notification is to their advantage because if they do partner notification, they will be able to make more sales. After all, the other partners will also be paying… I believe in my view that will be able to support it.” Male, Healthcare administrator 2

#### Domaine 3: community-based STI PN model

*Theme 1: Expanding the definition of community for STI care delivery.*

When asked about community-based STI PN models, most participants focused on village health teams [(VHTs) also known as community health workers], and some participants discussed other groups such as the religious leaders, politicians, civil society organizations, and traditional healers as key stakeholders in STI care. These groups were viewed as an important part of partnered couples' support network and a source of counseling.

“Here we have to look at people who do sex counselling [Interruption] then if you are going for marriage, it is the church so religious leaders should be part. Of course, politicians do everything they need to be part too. You need to look at the health workers and we need to look at the community opinion leaders and activists like women groups and civil society organizations.” Male, Healthcare administrator 3

“Like the chairman being involved in it [STI partner notification], the chairman then calls counselors to come in the area to teach the people… Elders of clans, leaders of villages have the experience to do that also.” Male, Patient 3

Participants noted that many patients visit traditional health providers as opposed to conventional medical providers. They outlined the need to partner with traditional healers so that they can guide patients on when to seek out STI testing and conventional medication.

“Involving traditional health providers just like these pregnant women drink herbs so that their children are not born with syphilis, but they won’t test because there are no resources…They will only settle for herbs, so if they can invite the herbalist to teach them the signs of all these STIs. That can guide our clients so it’s also good if they can find the services that they are looking for.” Male, Healthcare worker 4

*Theme 2: Local VHTs for STI PN may not uphold confidentiality while non-local VHTs may be more welcome.*

Some participants across all four participant groups were skeptical of discussing STIs and other potentially stigmatizing conditions with VHTs. They worried that VHTs were not professionally trained and therefore would not respect patients' confidentiality. They also noted that local VHTs were regular community members with whom they likely had a connection and history. This familiarity made participants wary of depending on VHTs for STI counseling.

“You see a VHT is my neighbor. So, my neighbor knows that I got an STI, but he also knows that I have a wife. They will ask questions. You get me? We have won that trust as health workers because we are distant from these people [patients]…There is that kind of confidentiality they want to preserve.” Male, Pharmacist 3

A few participants noted that patients could be more open to obtaining counseling from VHTs if they were not local to a community and a stranger. Having outsiders work in a community could bypass the anticipated stigma of a neighbor knowing about a private health condition and promote patient's receptivity to working with VHTs.

“In the VHT system you will find that a VHT cannot visit a certain family. They will say that we cannot be visited by our neighbors whom we have been fighting with. That is the challenge of having VHTs who have been living there for centuries…Sometimes those models can work if there are hired people, non-residents who come in to do community work and not the residents.” Male, Healthcare administrator 3

*Theme 3: VHTs should focus on advocacy and education.*

The same participants who worried about confidentiality also worried about VHTs not having the training and education of a healthcare worker, and therefore should have a limited role in caring for patients. These participants preferred patients to be evaluated, treated, and counseled by trained healthcare practitioners.

“These people delivering information are not health workers. There are [topic] areas where they are not supposed to go. If they [patients] come to the hospital, they can get the rest [of the information], so sensitizing them and training them. There’s information that they [VHTs] shouldn’t give because they should have a limit.” Male, Healthcare worker 5

Others thought VHTs were appropriate to provide STI PN counseling and emphasized that VHTs should focus on sensitization (education) and advocacy as opposed to treatment. Generally, participants were unfamiliar and uncomfortable with VHTs providing medications or treatment for medical issues.

“The role they [VHTs] have is for sensitization, to make sure that people do not get involved in bad acts, for example, sleeping around.” Male, Patient 3

“They [VHTs] also have a role to play. Sensitization and advocacy yes, but treatment and follow-up no.” Male, Pharmacist 3

#### Domaine 4: healthcare system tools for STI PN

*Theme 1: There is a tension between the ideal scenario of evaluating and testing a patient's partner for STIs with the real-world practicalities of PDPM.*

Participants noted several concerns regarding PDPM. They believed that prescribing medications to partners who had not been evaluated and diagnosed with an STI by a healthcare provider was inappropriate and could lead to antimicrobial resistance. They also worried that partners would refuse to take medications without an official STI diagnosis, especially if they are asymptomatic and feeling well. Additionally, participants noted that prescribing medications to partners could be cost-prohibitive for some patients; such practices could also lead to antibiotic stock-outs for people who have actually been diagnosed with an STI. Finally, healthcare providers voiced a frustration with not knowing the outcome for a patient and their partner after prescribing PDPM and preferred to evaluate the partner themselves.

“A man might ask you why you took for him that medicine and who told you that he was sick. That is an important issue. He will even refuse to take the medicine compared to when you go with him to the hospital.” Female, Patient 4

“In the public health centers like here we force partner treatment and we’ve been packing drugs for them so at times we are not sure if the partners accept to take those drugs. So now with the scarcity of the drugs, its challenging to say, ‘take the drugs to your partner’ so it becomes expensive for someone to treat all his key partners.” Male, Healthcare worker 4

Despite these concerns, approximately half the participants stated the benefits of PDPM for certain types of patients, especially those whose partners would not present to clinic for an evaluation. Reasons for not returning to clinic include a lack of funds for transport or long work-hours. While all participants noted they would ideally evaluate partners in person, many appreciated the utility and practicality of PDPM in the real-world context of limited resources and access to patients and partners.

“For STIs there is an assumption, a very good assumption, that if they are sleeping together, then the STI must have been passed. So, you could be maybe 90% sure that if you know that they have been sleeping together, then the other partner has the infection. So, it could be a good way because you know that drugs are going to move today rather than waiting on the other person to come and get screened, yet he might not turn up.” Male, Healthcare administrator 3

*Theme 2: STI PN tools that rely on phones are not yet practical for the general patient population.*

Participants noted several issues with STI PN methods that relied on phones, such as phone calls, text messages, and phone applications. One major issue is the fact that many people in Uganda do not own phones and when they do own phones, access to cellular data for phone calls and/or the internet can be limited. Additionally, many people are illiterate, precluding their use of text messaging or applications.

“I do not know what the statistics say but not all Ugandans own phones. I do not know how many Ugandans own a phone or can read and write.” Male, Pharmacist 1

Further, when people do own phones, they often share a single phone among family members. Even when an individual does not share a phone, there are still concerns about privacy as participants feared text messages concerning potentially stigmatizing issues, like STIs, being viewed by others.

“You are sending a message to someone but you are not sure that that person is the one who will receive that message… It could be the cause of problems in a marriage because the intended recipient for the message accidentally happened not to have been the one.” Male, Healthcare administrator 2

Participants also noted pervasive mistrust in regards to text messages and phone calls, citing scammers and imposters using the phone for deception. Most participants preferred in-person communication or communicating STIs with the aid of healthcare workers.

“You can send it and he will become like fire, very angry and heated up…so as a woman, wait for your man to come back home and sit then talk to him about it.” Female, Patient 1

Participants disliked the concept of anonymous messaging applications, as they reported that in a culture dominated by married, heterosexual relationships, such anonymous notification would not be anonymous for people who were monogamous with their partners.

“I personally don’t think [anonymous messages are] good because not everyone will take those messages serious. Another thing is that it might create family problems because if I receive a text message from someone I don’t know that I might have been exposed to an STI, and I know I have personally not slept with any other person except my partner, I will immediately know that my one partner is cheating.” Male, Pharmacist 5

#### Domaine 5: healthcare system needs to facilitate STI PN

*Theme 1: There is mistrust among healthcare fields due to heterogeneity of practice and the need for standardization.*

Participants were generally skeptical of other providers' ability to engage in appropriate STI care. They sometimes believed other professions to be unknowledgeable, untrustworthy, and financially motivated in regards to STI care.

“A patient comes for a test, then he’s tested negative but they [clinicians] will write positive and write drugs for him in a language he can’t understand so that they make him buy drugs and then they get money.” Female, Healthcare worker 3

Some of this mistrust may be due to the wide array of providers, from pharmacists to laboratory technicians to counselors, all caring for people with STIs. Participants noted that providers have varied levels and types of training leading to a broad range of STI knowledge and practice styles which were not always in line with the standard-of-care. The disparate practice styles in a fractured STI care context may lead some providers to mistrust the ability of their colleagues in different healthcare fields to engage in appropriate STI care.

“There is a very huge gap to achieve what you are trying to say because there is no board that unites all those that you are talking about, instead they are separate. Hospitals have their different forums and pharmacies have their own, drug shops the same, so there is no forum that joins these together. Each facility is handling STI partner notification differently.” Male, Pharmacist 6

“Not everyone is testing and treating; some are giving medicine according to the symptoms. If they assess and find that the symptoms are more, then they’ll give you medication without testing, but if they don’t show they do urinalysis and maybe a CBC [complete blood count].” Female, Pharmacist 7

Participants outlined that reduced STI practice variation among healthcare fields is needed to overcome their divergent practice styles. Potential solutions included creating roles for people to link different levels of care from local to tertiary as well as different types of care from the community to medical facilities.

“Establish STI focal persons at the facilities so that they may become a bridge between the communities, facilities, and maybe our regional referral hospital since there is an STI department. We know that if someone has a case that is very stubborn, we refer to the nearby regional referral but such a system is not there.” Male, Healthcare administrator 4

Others suggested that healthcare fields need clearer regulations and methods of enforcement across public and private sectors to ensure consistency in care.

“I cannot really say much about the private sector…They are professional, but sometimes they need serious supervision to make sure they adhere to the standards.. The way I do it here should be the same way they do it, but it might not be like that all the time so they need to be supported. The standards exist but anyway, adhering to them is another matter and enforcing them is another matter.” Male, Healthcare worker 6

*Theme 2: Care should be tailored to patient needs.*

Participants emphasized the importance of initially accessing local care prior to resorting to higher levels of care which are often inconvenient and costly. They highlighted the opportunity to work with local, lower-level health centers and additional methods of engaging with community care.

“Most STIs are not life-threatening conditions if they are managed early. The best way to manage diseases is at community level and at the health centers that are in the community. You do not want to have STIs managed at a regional referral hospital so it’s a system that should be strengthened in the community health centers.” Male, Healthcare administrator 3

Care should ideally be tailored for the specific needs of each patient. Certain methods and tools will work best for certain people, and providers should be able to work with patients to design the best STI PN method for each person.

“I think we can put a lot of things on table as a menu that if you feel you cannot talk to your partner, what means of communication can we use? Then people will tell you. Whoever suggests a phone, you use a phone, whoever suggests a home visit you use it. You use whatever people suggest. I think people should look at it as not only one, but it should be a number of approaches such that someone will pick and say that this method works for me.” Male, Healthcare worker 6

## Discussion

In this qualitative assessment of STI PN models, patient and healthcare provider participants in rural, southwestern Uganda felt each model (nursing-, pharmacy-, and community-based) had unique attributes; they also identified informative healthcare system tools and needs to facilitate partner notification. In general, patient and healthcare provider participants were aligned in their opinions on STI models and tools. The nursing model was well-liked as nurses generally are trusted to perform a variety of tasks, though a patient participant strongly noted a bad experience with a nurse and resulting distrust. Pharmacies are already an early point of contact for many patients, and pharmacy monetary incentives are aligned with the goal of STI PN inclusive of PDPM. Participants outlined solutions to pharmacy model barriers, such as creating private spaces and providing pharmacists with STI counseling training. For a community-based model, participants focused on VHTs and suggested bringing in outsiders as opposed to locals to serve as VHTs, given the potential stigma associated with STIs. They also noted that VHTs should focus on education and advocacy and did not think VHTs could dispense medication. Regarding STI PN tools, participants felt tension between the ideal of partner evaluation and the real-world benefits of PDPM when uncertain that partners would engage in care. Participants also were skeptical and unfamiliar with phone-related communication such as voice messages, text messages, and phone applications. There was general mistrust of healthcare providers in different fields to manage STIs appropriately which likely stems from the range of healthcare provider trainings and perspectives. Participants noted that greater standards and regulations are needed to align healthcare providers. In addition, participants wanted flexibility to tailor STI PN to patient needs through shared decision-making between provider and patient.

Nurses were perceived as professional, trustworthy, and appropriate providers to counsel and assist patients with STI PN, though a dissenting patient voice found that nurses could be rude. Despite nurses being integral to STI testing, counseling, and education, relatively little research has focused on the roles of nurses in STI PN with most research taking place in resource-rich settings. Several US-based studies found that nursing interventions focused on counseling through phone calls, text messages, and home visits could lead to increased STI PN and treatment among adolescents and young adults (13, 14). Nursing is often the default occupation in task-shifting models due to their varied skills, however this reliance can result in nursing burn-out and decreased patient satisfaction. A Dutch qualitative study exploring STI PN barriers and facilitators among 22 nurses found that barriers included sub-optimal STI PN guidelines, a belief that STI PN was the responsibility of the patient, and a lack of feedback regarding the outcomes of their counseling and STI PN (15). Training in motivational interviewing served as a facilitator. Nursing-based motivational interviewing has shown to be effective for STI PN, allowing more tailored counseling and nurses and patient to take shared-responsibility for the patient's behavior and intentions (16). However, nurses noted that this technique often requires more personal investment. Additionally, stigma reduction measures are crucial for all healthcare professions working with patients affected by stigma-related conditions. Nursing-based STI PN interventions will require balancing increasing our demands on nurses with increasing methods to support nurses inclusive of trainings focused on patient-centered care in an effort to increase patient satisfaction.

Participants felt that the pharmacy-based model is inherently designed to support STI PN in the Ugandan setting as pharmacies are monetarily incentivized to support STI PN and PDPM and are also early points of contact for patients seeking care. A cross-sectional study of 50 pharmacies in the Kibera slum of Nairobi, Kenya, found that one-third of patients went to pharmacies as their first point of contact for care and two-thirds of patients did not have prescriptions for specific medications, confirming our findings (17). Notably, research in East Africa demonstrates that the medications dispensed from pharmacies may or may not correspond with the local Ministry of Health syndromic management guidelines, ranging from 10% to 90% correspondance (17, 18). This variability in appropriate medications dispensed indicates variable accuracy in diagnosis and has implications for whether appropriate PDPM is dispensed. In this same Kenyan study, two male mystery shoppers presented to participating study pharmacies with symptoms consistent with gonorrhea or genital ulcer disease (17). All but one pharmacy staff offered STI counseling, and 84% counseled specifically on partner treatment, suggesting that pharmacy staff are often motivated and would benefit from further training to increase their role in STI care. Additionally, our study participants outlined that space and training were needed for this model to adequately support STI PN. Qualitative work from resource-rich settings have also found that a lack of space and privacy are major barriers to integrating STI counseling into pharmacies (19, 20). These issues will be all the more pivotal when thinking through pharmacy-based care as STI point-of-care diagnostics are developed and disseminated in RLS (19). Retraining pharmacy staff and redesigning pharmacies to ensure privacy and facilitate counseling could better support their role in in STI care.

Per our study participants, an ideal community-based model would focus on VHTs educating and advocating for patients. However, participants outlined threats to confidentiality within the community-based VHTs model. This lack of trust around VHT confidentiality is also described in studies related to HIV disclosure (21). Community health worker services in Uganda were first developed a in 2001 (22) and based on the natural helper model (23). This model relies on the premise that within every community there are informal helping networks; certain people are naturally trusted and inclined to provide social support. To further explore this concept, a qualitative, ethnographic study in Uganda's Luwero district with community members and VHTs found that the VHT selection process neglected the natural helper model which led to community distrust, the VHTs feeling alienated, and the VHTs subsequently losing morale for their work (24). A lack of trust in community health workers in our setting may be similarly due to a disconnect with the natural helper model. To overcome these issues, some of our participants suggested introducing VHTs from outside communities, though this idea challenges the Ugandan Ministry of Health's criteria for VHT selection (25). Additionally, our participants were reluctant to allow VHTs to dispense medications to patients, though VHTs routinely dispense medications such as anti-malarials and antibiotics for respiratory infections. Further exacerbating these issues is the lack of compensation and resources that VHTs receive for their work (26). A discrete choice experiment among 399 VHTs in 8 districts across Uganda found that transportation was the most important incentive for VHTs, indicating that even small support investments can improve working conditions and likely morale (27). Ensuring that VHTs are actually selected by the community and that VHTs are provided compensation for their efforts will be crucial for continuing to rely on this group of workers for expanding healthcare in Uganda and similar settings.

STI PN tools were met with uncertainty among our participants as most lacked personal experience with these tools. Our participants had mixed feelings on PDPM with nearly all participants favoring partner evaluation and some participants stating the benefit of PDPM in situations where a partner would not be evaluated by a healthcare provider. A randomized controlled trial of 383 participants in Kampala, Uganda found a statistically significant risk ratio of 2.4 for partner treatment when comparing participants assigned to PDPM compared to patient-based referral, indicating the feasibility of this approach (28). A subset of PDPM, called accelerated partner therapy, in which partners are evaluated by a healthcare provider either in-person or via phone was consistent with our participants' preferences. Accelerated partner therapy models have similarly been explored in the UK with general providers preferring accelerated partner therapy hotlines over pharmacy-based accelerated partner therapy models (29). Our participants also considered tools such as PDPM and PN messaging applications were considered if these tools could supplement but not replace direct healthcare provider support. Qualitative research from South Africa found such STI PN tools may most directly benefit patients with low health literacy as these patients specifically had limited STI diagnosis recall which could impede their ability to engage in PN (30). While technological advancements have the potential to aid in STI PN, these tools will need to be adapted to various RLS contexts to enhance their application and uptake.

In many RLS, different types of healthcare provider with different backgrounds and education care for patients with STIs ranging from physicians to pharmacists to laboratory technicians. This variety of training and perspectives leads to different care practices across the field. This issue is likely exacerbated by the fact that most RLS do not have access to gold standard diagnostics, though suboptimal tests are available (31). Our participants voiced differing STI care beliefs based on their background. For example, some participants believed testing was crucial while others based their care on syndromic management. This mistrust spans the entirety of the STI care continuum from ideas about STI transmission to diagnostics to counseling. Access to evidence-based resources in addition to strengthening the capacity of healthcare provider is crucial to achieving quality, standardized care (32). Routine introductory and refresher trainings on the standard-of-care in the local setting and on the perspectives of various healthcare providers could standardize practice and increase healthcare providers’ respect for other healthcare fields. Additionally, the dissemination of point-of-care diagnostics in RLS may further bolster the standardization of STI care practices (33).

### Limitations

This study balances both limitations and strengths. A diverse group of STI care stakeholders participated in interviews including healthcare providers as well as patients. Groups varied in expansiveness with healthcare workers encompassing a wide array of providers such as doctors, nurses, and community health workers compared to the more homogenous group of pharmacists. The small group numbers limited our ability to analyze the data by specific healthcare provider type. However, this diversity allowed our team to access an array of perspectives on STI PN while balancing the limitations of the research timeline. The candidate STI PN models we presented potentially limited our participants' creativity when brainstorming novel methods to support STI PN, though these models also anchored our conversations, aiding in participant discussions of potentially abstract ideas. Finally, participants had difficulty discussing STI PN in isolation, and often expanded their points to STI care in general.

## Conclusion

RLS have traditionally lacked capacity to support STI PN. In this analysis we explored STI stakeholders' thoughts on three candidate STI PN models as well as PN tools. We found that all three candidate STI PN models (nursing-based, pharmacy-based, and community-based) had aspects that made them feasible for STI counseling and care. Nurses were generally trusted to carry out numerous different roles, though one participant did have negative counseling experiences with nurses. The pharmacy model was a convenient and logical point of contact for STI PN, though restructuring of the physical space and pharmacy staff training was required. Participants focused their definition of community on VHTs and thought non-local VHTs may be more trust worthy to maintain the confidentiality of stigmatizing issues. Finally, participants thought VHTs should focus on advocacy and education and refrain from dispensing medications. Overall, the STI care stakeholders are diverse and increased integration and coordination is needed to ensure streamlined care. The data gleaned from this work will inform a Delphi method where similar stakeholders will further refine an STI PN delivery services model for future development and testing. Given the high STI incidence globally and specifically in sub-Saharan Africa, continuing to explore innovative STI care models that resonate with patients and healthcare practitioners will be all the more important.

## Data Availability

The datasets presented in this article are not readily available because these data include qualitative interviews with potential identifiers. We did not obtain participant permission to share interview transcripts during the informed consent process. We may be able to share some data with certain people once de-identified. Requests to access the datasets should be directed to pchitneni@mgh.harvard.edu.

## References

[1] World Health Organization. Global Progress Report on HIV, Viral Hepatitis and Sexually Transmitted Infections, 2021. Accountability for the Global Health Sector Strategies 2016–2021: Actions for Impact. Geneva: World Health Organization (2021).

[2] World Health Organization. Guidelines for the Management of Symptomatic Sexually Transmitted Infections. Geneva: The World Health Organization (2021). 34370424

[3] World Health Organization. Guidelines for the Management of Sexually Transmitted Infections. Geneva: World Health Organization (2001).

[4] Ministry of Health Uganda. Uganda Clinical Guidelines 2016: National Guidelines for Management of Common Conditions. Kampala: Ministry of Health Uganda (2016).

[5] Ferreira A Young T Mathews C Zunza M Low N. Strategies for partner notification for sexually transmitted infections, including HIV. Cochrane Database Syst Rev. (2013) 10:CD002843. 10.1002/14651858.CD002843.pub2 PMC7138045 24092529

[6] Taleghani S Joseph-Davey D West SB Klausner HJ Wynn A Klausner JD. Acceptability and efficacy of partner notification for curable sexually transmitted infections in sub-Saharan Africa: a systematic review. Int J Std Aids. (2019) 30(3):292–303. 10.1177/0956462418803983 30396318 PMC6441466

[7] Chitneni P Owembabazi M Kanini E Mwima S Bwana MB Psaros C STI partner notification goals, strategies, and outcomes among women and men in HIV-serodifferent relationships with recent or planned pregnancy in rural southwestern Uganda [Oral abstract]. In: Inter-CFAR Women and HIV Symposium, Center for AIDS Research (CFAR); 2021 Oct 12–13; Virtual (via Zoom). (2021).

[8] Damschroder LJ Aron DC Keith RE Kirsh SR Alexander JA Lowery JC. Fostering implementation of health services research findings into practice: a consolidated framework for advancing implementation science. Implement Sci. (2009) 4:50. 10.1186/1748-5908-4-50 19664226 PMC2736161

[9] Tong A Sainsbury P Craig J. Consolidated criteria for reporting qualitative research (COREQ): a 32-item checklist for interviews and focus groups. Int J Qual Health Care. (2007) 19(6):349–57. 10.1093/intqhc/mzm042 17872937

[10] Callaghan M Ford N Schneider H. A systematic review of task- shifting for HIV treatment and care in Africa. Hum Resour Health. (2010) 8:8. 10.1186/1478-4491-8-8 20356363 PMC2873343

[11] Braun V Clarke V. Successful Qualitative Research: A Practical Guide for Beginners. London: Sage Publiations (2013).

[12] Chaudoir SR Fisher JD. The disclosure processes model: understanding disclosure decision making and postdisclosure outcomes among people living with a concealable stigmatized identity. Psychol Bull. (2010) 136(2):236–56. 10.1037/a0018193 20192562 PMC2922991

[13] Ha MM Belcher HME Butz AM Perin J Matson PA Trent M. Partner notification, treatment, and subsequent condom use after pelvic inflammatory disease: implications for dyadic intervention with Urban youth. Clin Pediatr (Phila). (2019) 58(11–12):1271–6. 10.1177/0009922819852979 31165630 PMC6868422

[14] Richards MJ Bogart A Sheeder J. Reducing barriers for expedited partner treatment in adolescents and young adults: a quality improvement initiative. Sex Transm Dis. (2024) 51(5):325–30. 10.1097/OLQ.0000000000001933 38301630

[15] Theunissen KA Schipper P Hoebe CJ Crutzen R Kok G Dukers-Muijrers NH. Barriers to and facilitators of partner notification for chlamydia trachomatis among health care professionals. BMC Health Serv Res. (2014) 14:647. 10.1186/s12913-014-0647-5 25526679 PMC4279885

[16] Kuyper L de Wit J Heijman T Fennema H van Bergen J Vanwesenbeeck I. Influencing risk behavior of sexually transmitted infection clinic visitors: efficacy of a new methodology of motivational preventive counseling. AIDS Patient Care STDS. (2009) 23(6):423–31. 10.1089/apc.2008.0144 19415987

[17] Kwena Z Sharma A Wamae N Muga C Bukusi E. Provider characteristics among staff providing care to sexually transmitted infection self-medicating patients in retail pharmacies in Kibera slum, Nairobi, Kenya. Sex Transm Dis. (2008) 35(5):480–3. 10.1097/OLQ.0b013e3181644b84 18360315

[18] Viberg N Mujinja P Kalala W Kumaranayake L Vyas S Tomson G STI management in Tanzanian private drugstores: practices and roles of drug sellers. Sex Transm Infect. (2009) 85(4):300–7. 10.1136/sti.2008.032888 19174423

[19] Baraitser P Pearce V Holmes J Horne N Boynton PM. Chlamydia testing in community pharmacies: evaluation of a feasibility pilot in south east London. Qual Saf Health Care. (2007) 16(4):303–7. 10.1136/qshc.2006.020883 17693680 PMC2464947

[20] Wood H Hall C Ioppolo E Ioppolo R Scacchia E Clifford R Barriers and facilitators of partner treatment of Chlamydia: a qualitative investigation with prescribers and community pharmacists. Pharmacy (Basel). (2018) 6(1):17. 10.3390/pharmacy6010017 29419807 PMC5874556

[21] Lukyamuzi Z Ssuna B Mirembe RN Mawanda D Kinkumu P Nalugo C Experiences and challenges of using community health worker-led mechanism in supporting HIV disclosure among adults living with HIV in heterosexual relationships in the rural Uganda. AIDS Res Ther. (2023) 20(1):14. 10.1186/s12981-023-00508-0 36906557 PMC10008611

[22] Uganda Bureau of Statistics. Uganda demographic and health survey 2016 (2016).

[23] Pancoast DL Chapman NJ. Roles for Informal Helpers in the Delivery of Human Services. Community Support Systems and Mental Health. New York: Springer (1982).

[24] Turinawe EB Rwemisisi JT Musinguzi LK de Groot M Muhangi D de Vries DH Selection and performance of village health teams (VHTs) in Uganda: lessons from the natural helper model of health promotion. Hum Resour Health. (2015) 13:73. 10.1186/s12960-015-0074-7 26346431 PMC4562111

[25] Uganda Ministry of Health. Village health team: strategy and operational guidelines (2010).

[26] Mays DC O'Neil EJ Jr Mworozi EA Lough BJ Tabb ZJ Whitlock AE Supporting and retaining village health teams: an assessment of a community health worker program in two Ugandan districts. Int J Equity Health. (2017) 16(1):129. 10.1186/s12939-017-0619-6 28728553 PMC5520299

[27] Agarwal S Tweheyo R Pandya S Obuya E Kiyomoto A Mitra P Impact of a recognition package as an incentive to strengthen the motivation, performance, and retention of village health teams in Uganda: a study protocol for a cluster randomized controlled trial. Trials. (2023) 24(1):428. 10.1186/s13063-023-07426-6 37353798 PMC10288687

[28] Nuwaha F Kambugu F Nsubuga PS Hojer B Faxelid E. Efficacy of patient-delivered partner medication in the treatment of sexual partners in Uganda. Sex Transm Dis. (2001) 28(2):105–10. 10.1097/00007435-200102000-00008 11234783

[29] Shackleton T Sutcliffe L Estcourt C. Is accelerated partner therapy partner notification for sexually transmissible infections acceptable and feasible in general practice? Sex Health. (2011) 8(1):17–22. 10.1071/SH10031 21371378

[30] Medina-Marino A Glockner K Grew E De Vos L Olivier D Klausner J The role of trust and health literacy in nurse-delivered point-of-care STI testing for pregnant women living with HIV, tshwane district, South Africa. BMC Public Health. (2020) 20(1):577. 10.1186/s12889-020-08689-3 32345293 PMC7189538

[31] Chitneni P Owembabazi M Muyindike W Asiimwe S Masete G Mbalibulha Y Sexually transmitted infection point-of-care testing in resource-limited settings: a narrative review guided by an implementation framework. Sex Transm Dis. (2023) 50(10):e11–6. 10.1097/OLQ.0000000000001848 37433000 PMC10527944

[32] Nambiar B Hargreaves DS Morroni C Heys M Crowe S Pagel C Improving health-care quality in resource-poor settings. Bull World Health Organ. (2017) 95(1):76–8. 10.2471/BLT.16.170803 28053367 PMC5180347

[33] Murtagh MM. The Point-of-Care Diagnostic Landscape for Sexually Transmitted Infections (STIs). Geneva: World Health Organization (2019).

